# Clinical outcomes of a combined transcatheter and minimally invasive atrial septal defect repair program using a 'Heart Team' approach

**DOI:** 10.1186/s13019-018-0701-1

**Published:** 2018-01-18

**Authors:** Shahrukh N. Bakar, Daniel J. P. Burns, Pantelis Diamantouros, Kumar Sridhar, Bob Kiaii, Michael W. A. Chu

**Affiliations:** 1 Division of Cardiology, Department of Medicine, Western University, Lawson Health Research Institute, 339 Windermere Road, London, ON N6A 5A5 Canada; 2 Division of Cardiac Surgery, Department of Surgery, Western University, Lawson Health Research Institute, London, ON Canada

**Keywords:** Atrial Septal defect, Minimally invasive, Transcatheter, Percutaneous

## Abstract

**Background:**

Contemporary transcatheter and minimally invasive approaches allow for improved cosmesis and eliminate sternotomy; however, access to a ‘Heart Team’ approach to minimally invasive atrial septal defect (ASD) repair remains limited in Canada.

**Methods:**

Retrospective chart review of all minimally invasive atrial septal defect repairs performed between 2009 and 2017 at a quaternary cardiac care centre were included. We compared residual shunt, functional status, periprocedural complications, and hospital lengths-of-stay between patients undergoing transcatheter and minimally invasive endoscopic ASD repair.

**Results:**

Between 2009 and 2017, 61 consecutive patients underwent ASD repair at a single centre: 28 patients underwent transcatheter closure (64.3% female; median age 57, interquartile range 43–70.5) and 33 patients underwent minimally invasive endoscopic repair (72.7% female; median age 37, interquartile range 24–50). Patient demographics were similar between the two groups with the exception of transcatheter patients having smaller defect size (1.65 cm versus 2.35 cm, *p* = 0.002). Procedural success was 93% (26/28) and 100% (33/33) for transcatheter and minimally invasive groups (*p* = 0.21), respectively. Periprocedural complications were similarly low between the two groups with the exception of longer hospital length-of-stay in the surgical patients (5 days vs 1 day, *p* < 0.0001). Over a follow-up period (transcatheter: 0.5–56.5 months, surgical: 0.25–89 months), there was no difference in residual shunt (14.3% versus 6.1%, *p* = 0.4) or NYHA I Functional Class (88.5% versus 96.9%, *p* = 0.21).

**Conclusion:**

Transcatheter and minimally invasive approaches to ASD repair are safe and feasible in selected patients using a ‘Heart Team’ approach and represent attractive alternatives to median sternotomy.

## Brief Summary

Atrial septal defects have traditionally been surgically corrected using a median sternotomy approach. Contemporary transcatheter and minimally invasive approaches allow for less invasive atrial septal defect repair with  improved cosmesis and eliminate the need for median sternotomy. Clinical outcomes of a Canadian multidisciplinary ‘Heart Team’ atrial septal defect repair program are presented.

## Background

Repair of atrial septal defects (ASD) have traditionally been performed through median sternotomy for many decades, however contemporary practice includes transcatheter-based approaches and minimally invasive endoscopic mini-thoracotomy approaches [1–3]. Long-term data show that median sternotomy repair of ASD is effective [4]. Catheter-based approaches have the benefit of no surgical scar, but require ongoing antiplatelet therapy and favourable anatomy for procedural success [5]. Minimally invasive endoscopic mini-thoracotomy has previously been shown to improve cosmesis with similar outcomes as median sternotomy for ASD closure [6–10]. Although research into minimally invasive surgical approaches is being actively pursued, access to minimally invasive approaches is limited to select centers of excellence in Canada [11]. A hybrid approach involving both cardiac surgery and interventional cardiology expertise is emerging as the preferred strategy for minimally invasive intervention [11, 12]. At our centre in London, Ontario, Canada, ASD repair is evaluated by a combined multidisciplinary team approach involving both interventional cardiology and cardiac surgery services since 2009. We present our single centre ‘Heart Team’ experience comparing early and late clinical outcomes of transcatheter device closure and mini-thoracotomy ASD repair in a Canadian setting.

## Methods

### Patient population and study design

All patients who had undergone minimally invasive atrial septal defect (ASD) closure from 2009 to 2017 were included in the study population. The inclusion dates were chosen based on the time when both therapies were available at our institution, and a multidisciplinary approach to minimally invasive closure had been formalized. All patients were initially evaluated by the London Structural Heart Team (interventional cardiology, echocardiography and cardiac surgery) to determine if a transcatheter option was feasible based of defect type, size, and morphology. Those patients deemed appropriate were planned for transcatheter device closure. In those patients unable to undergo device closure, or in those who failed device closure, a surgical referral was made to undergo ASD pericardial patch repair by either a 3–4-cm right anterolateral mini-thoracotomy (*n* = 26) or right peri-areolar approach (*n* = 7). Defects considered inappropriate for device closure were: larger than 38 mm in diameter, non-secundum defects, secundum defects with insufficient tissue rims, multiple defects thought better closed surgically, and “Swiss cheese septum” type defects. Patients were allowed a choice of therapy if both were considered equivalent by the multidisciplinary cardiac care team, which included both interventional cardiology and cardiac surgery, in accordance with established guidelines [13].

Available data had been prospectively collected from the time of the patient’s procedure and kept in an institutional database. Data regarding a patient’s current clinical condition was taken from their most recent clinical follow-up. Those patients not followed within 1 year of this study were brought back for additional clinical follow-up including transthoracic echocardiography (TTE). The Health Sciences Research Ethics Board at Western University approved the study protocol.

### Outcomes

The primary outcome of interest was the presence of any residual intra-cardiac shunt at most recent follow-up. Secondary outcomes included the patient’s current functional status (indicated by the patient’s New York Heart Association (NYHA) class), presence of post-procedure headaches, and post procedure stroke or transient ischemic attack (TIA). Peri-procedural secondary outcomes included all cause mortality, stroke, myocardial infarction, infection, major bleeding, blood transfusion, and length of stay in hospital and intensive care. Categorical outcomes were recorded as a presence or absence of the condition, as documented in the institutional database, as well as the patient’s medical record.

### Statistical analysis

Normally distributed continuous variables were compared using a 2-sample t-test. Non-normally distributed continuous variables were compared using the non-parametric 2-sample Wilcoxon rank sum test. Binary variables were compared using the chi-squared test, or Fisher’s exact test if individual group cell numbers were fewer than 5. The unadjusted relationship between closure method and presence of any residual shunt was modelled using Kaplan-Meier time-to-event methods, with the 2 curves compared using the log-rank test statistic. This was repeated for the composite outcome of residual shunt greater than mild, device erosion, embolization, thrombosis, endocarditis, thromboembolism, or stroke. An adjusted analysis for presence of any residual shunt was performed using Cox proportional hazard modelling. Covariates included in the Cox model were age, sex, shunt fraction, defect size, and non-secundum defect. The proportional hazard assumption was tested by generation of log-log plots and by use of Schoenfeld residuals.

Confidence intervals were set at 95%; all *p* values were 2-sided and considered statistically significant if < 0.05. When possible, exact *p* values have been reported. All statistical analysis was performed using Stata 13.1 (StataCorp LP, College Station, TX, USA).

## Results

**Fig. 1:**
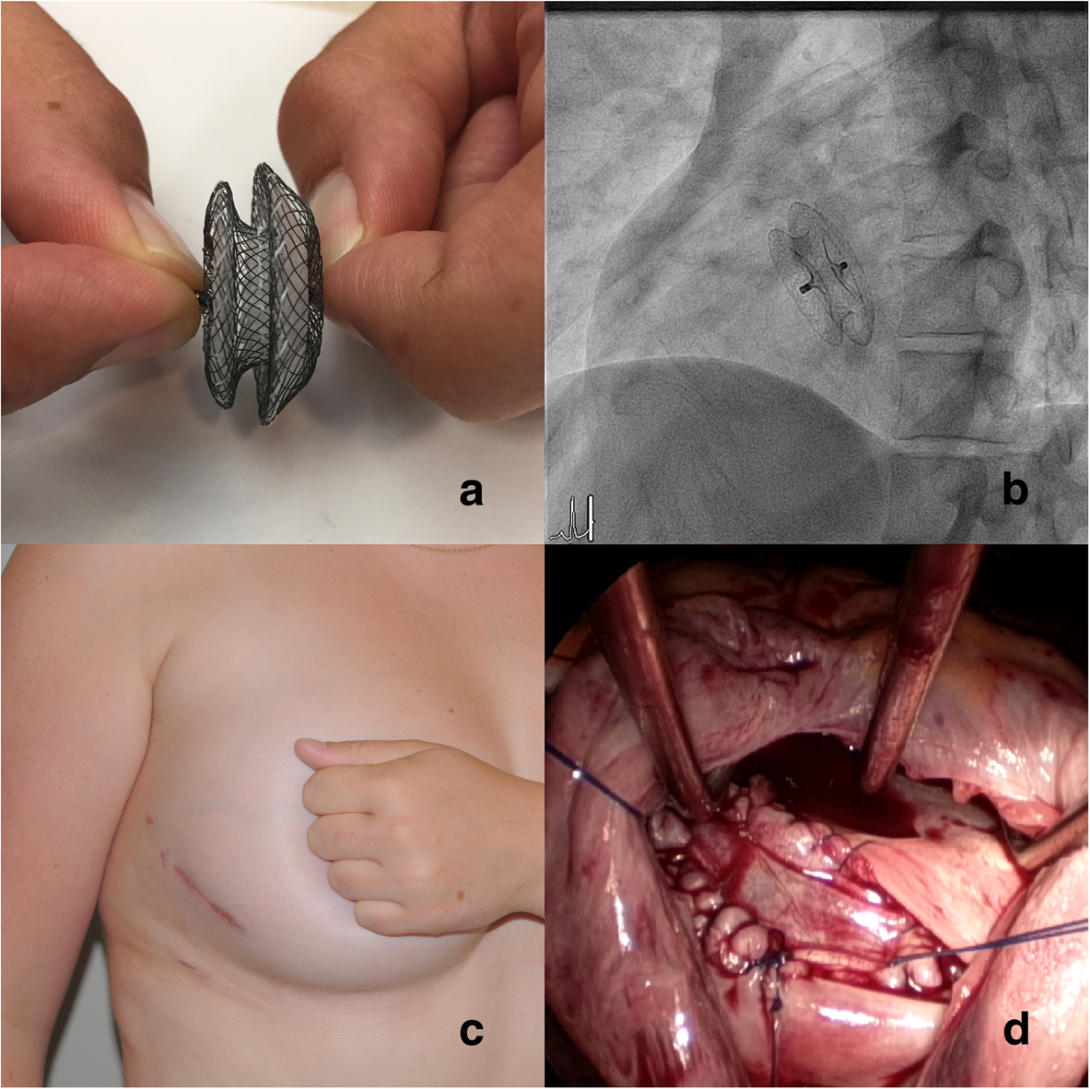
Panel **a** - Ex-vivo photograph of Amplatzer septal occluder device (St. Jude Medical, St. Paul, MN, USA). Panel **b** - Fluoroscopic view of deployed Amplatzer device. Panel **c** – Postoperative result of right mini-thoracotomy incision Panel (**d**) – Intraoperative view of atrial septum showing partially repaired septal defect

In total, 61 patients underwent a minimally invasive approach to ASD closure. Twenty-eight patents underwent transcatheter device-based closure, and 33 underwent surgical closure. Figure 1 shows an Amplatzer septal occluder device (St. Jude Medical, St. Paul, MN, USA) in Panel A, along with a fluoroscopic image of the device immediately after deployment (Panel B). Figure 1 also shows a typical right mini-thoracotomy incision in the post-operative setting, showing excellent cosmesis (Panel C) along with an intraoperative view of the autologous pericardial patch being sewn to close the ASD (Panel D).

Surgical patients were younger than the transcatheter group, with a median age of 37 versus 57 (*p* < 0.001). Surgical patients also had a lower overall body mass index (BMI), with a mean of 25 versus 28.2 (*p* = 0.01). Otherwise, preoperative patient characteristics were similar between groups, as shown in greater detail in Table 1.

**Table 1:** Baseline patient characteristics^a^

		Transcatheter (*n* = 28)	Surgical (*n* = 33)	*p* value
Age, median (IQR)		57 (43–70.5)	37 (24–50)	< 0.001
Female sex (%)		18 (64.3)	24 (72.7)	0.48
BMI, mean (SD)		28.2 (4.8)	25.0 (3.5)	0.01
NYHA	1	17 (60.7)	20 (60.6)	0.93
	2	10 (35.7)	11 (33.3)	
	3	1 (3.6)	2 (6.1)	
	4	0 (0)	0 (0)	
Hypertension		10 (35.7)	6 (18.2)	0.15
Diabetes		3 (10.7)	2 (6.1)	0.65
Dyslipidemia		9 (32.1)	6 (18.2)	0.24
Coronary disease		3 (10.7)	1 (3.0)	0.33
COPD		0 (0)	1 (3.0)	1
Stroke		6 (21.4)	6 (18.2)	0.76
CHF		1 (3.6)	2 (6.1)	1
CKD		0 (0)	0 (0)	
Qp:Qs, median (IQR)		2.0 (1.8–2.4)	2.25 (1.7–3.0)	0.37
RVSP (mmHg), median (IQR)		32.5 (25.5–42)	26 (24–30)	0.009

^a^Reported as *n*(%) unless otherwise specified

*IQR* Interquartile range, *SD* Standard deviation, *NYHA* New York Heart Association, *COPD* Chronic obstructive pulmonary disease, *CHF* Congestive heart failure, *CKD* Chronic kidney disease, *RVSP* Right ventricular systolic pressure

Predictably, all ASDs in the transcatheter group were secundum defects with the exception of a single patent foramen ovale (PFO). The surgical group contained 5 sinus venosus defects with partial anomalous pulmonary venous connections, 3 PFOs, 1 unroofed coronary sinus, and 1 partial atrioventricular septal defect (AVSD). Similarly, defect size was significantly larger in the surgical group (2.35 versus 1.65 cm, *p* = 0.002). There were 2 device failures, necessitating referral for surgical repair at similar rates as previously reported [14]. In the surgical group, 26 were approached through a right anterior mini-thoracotomy, while 7 were female patients who underwent a peri-areolar incision. Three patients underwent concomitant tricuspid valve repair, 2 patients underwent mitral valve repair, and 2 patients underwent cryoablation. Detailed procedural data is shown in Table 2.

**Table 2:** Procedural data^a^

		Transcatheter (n = 28)	Surgical (n = 33)	*p* value
Defect size (cm), median (IQR)		1.65 (1.25–2.0)	2.35 (1.9–2.8)	0.002
Defect type	Secundum	27 (96.4)	26 (78.8)	
	Sinus venosus		5 (15.2)	
	PFO	1 (3.6)	3 (9.1)	
	Partial AVSD		1 (3.0)	
	Unroofed CS		1 (3.0)	
Device	Amplatzer	21 (75.0)		
	Gore	6 (21.4)		
Device size (cm), median (IQR)		2.2 (1.9–2.6)		
Procedural Success		26 (92.8)	33 (100)	0.21
Additional	TV repair		3 (9.1)	
	MV repair		2 (6.1)	
	Ablation		2 (6.1)	
Approach	Mini-thoracotomy		26 (78.8)	
	Peri-areolar		7 (21.2)	
Bypass time (min), mean (SD)			122.8 (43.4)	
Cross clamp time (min), mean (SD)			69.9 (29.7)	

^a^Reported as *n*(%) unless otherwise specified

*IQR* Interquartile range, *SD* Standard deviation, *PFO* Patent foramen ovale, *AVSD* Atrioventricular septal defect,: Coronary sinus

The median hospital length-of-stay for the transcatheter group was 1 day, with no patient requiring admission to intensive care (ICU). Length of stay was longer in the surgical group with median ICU length of stay of 1 day, with a median hospital stay of 5 days (*p* = < 0.0001 for each versus the transcatheter group). Otherwise, no significant peri-procedural outcome differences were detected between the intervention groups. Five patients in each group experienced paroxysmal atrial fibrillation post-procedure. A single patient in the transcatheter group suffered a bleeding complication from the femoral puncture site that resolved with additional manual pressure and did not require blood product transfusion. A single patient in the surgical group received 2 units of packed red blood cells for an asymptomatic hemoglobin level below 70 g/L during the post-operative ICU stay. No patients required transfusion of additional products such as plasma, platelets, cryoprecipitate, or recombinant activated factor VII. Detailed results for peri-procedural outcomes are shown in Table 3.

**Table 3:** Peri-procedure outcomes^a^

	Transcatheter (*n* = 28)	Surgical (*n* = 33)	*p* value
ICU stay, median (IQR)	0 (0–0)	1 (1–1)	< 0.0001
Hospital stay, median (IQR)	1 (1–1)	5 (4–5)	< 0.0001
Reoperation - bleeding	0 (0)	0 (0)	
Death	0 (0)	0 (0)	
Myocardial infarction	0 (0)	0 (0)	
CVA	0 (0)	0 (0)	
IABP	0 (0)	0 (0)	
Arrest	0 (0)	0 (0)	
Infection	0 (0)	0 (0)	
Atrial fibrillation	5 (18.5)	5 (15.2)	0.74
Renal failure	0 (0)	0 (0)	
Ventilator dependence	0 (0)	0 (0)	
Bleeding	1 (3.7)	0 (0)	0.45
Blood product use	0	1 (1–1)	0.36

^a^Reported as n(%) unless otherwise specified

*IQR* Interquartile range, *u* Units, *PRBC* Packed red blood cells

**Fig. 2:**
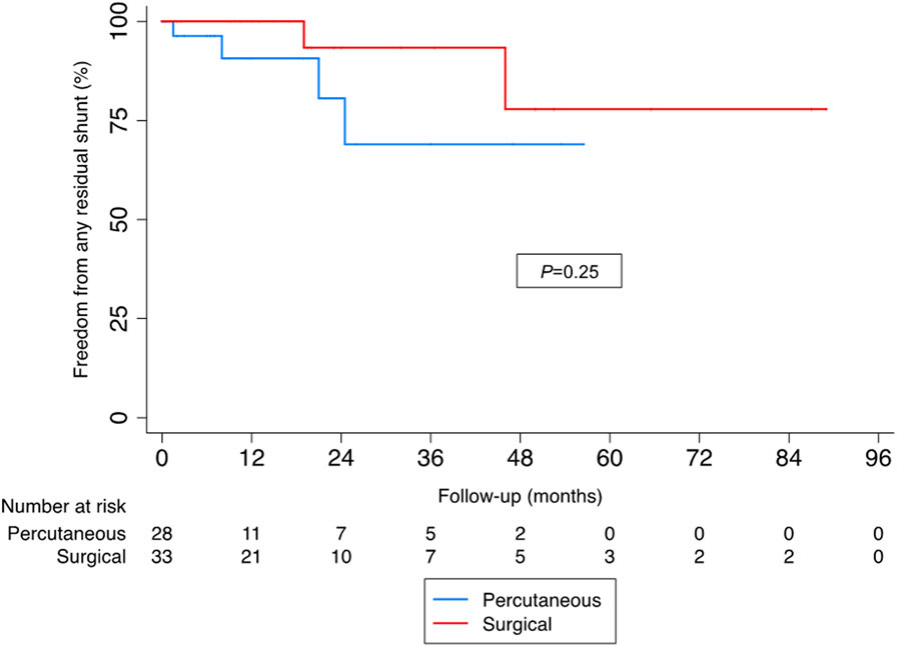
Time to event analysis for freedom from any residual shunt

**Fig. 3:**
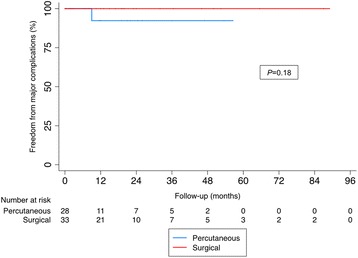
Time to event analysis for freedom from death and/or major complications.**Major complications was defined as significant residual shunt defined as greater than mild, device erosion, embolization, thrombosis, endocarditis, thromboembolism, or stroke

**Fig. 4:**
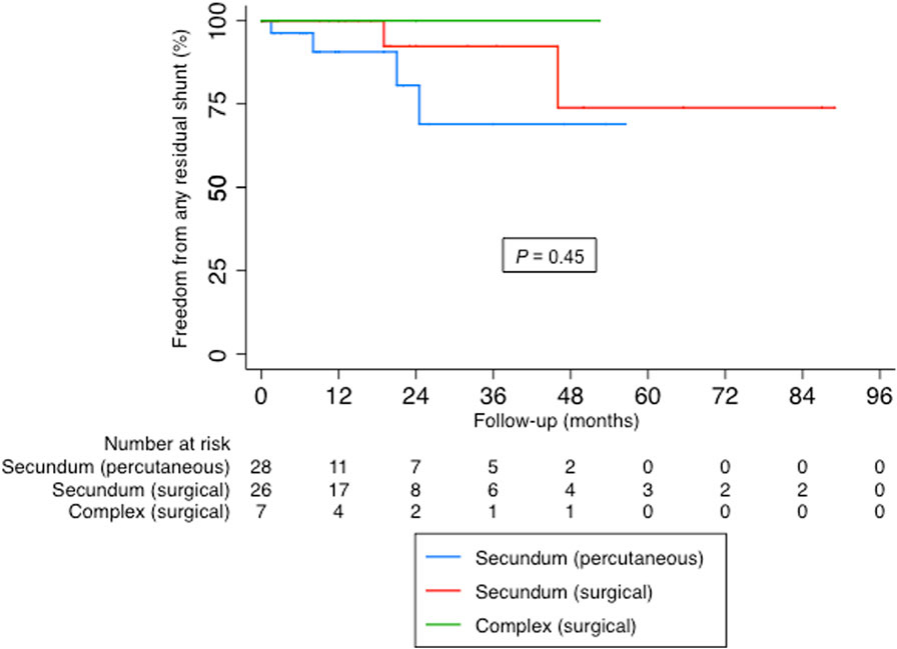
Time to event analysis for freedom from any residual shunt showing secundum versus complex intervention in the surgical group

Follow-up time ranged from 0.5–56.5 months in the transcatheter group and 0.3–89.0 months in the surgical groups. Median follow-up time was 8.3 months in the transcatheter group and 15 months in the surgical group (*p* = 0.3). Four residual shunts were identified on post-operative TTE versus 2 in the surgical group (*p* = 0.4). All residual shunts were asymptomatic, and graded as trace to mild. On Kaplan-Meier analysis, the 2 curves appeared divergent; however, they were not found to be significantly different by the log-rank test statistic (Fig. 2). The proportional hazard assumption was not violated. Log-log plots showed parallel curves, and the hazard function between groups was not statistically significant using Schoenfeld residuals (*p* = 0.27). The adjusted Cox proportional hazard model failed to show a significant difference in risk of residual shunt between groups (HR 0.41, 95% CI: 0.02–8.60). Detailed adjusted results of the multivariable Cox proportional hazard model are shown in Table 4. Similarly, there was no significant difference in late complications, defined as a composite of significant residual shunt (greater than mild on Doppler colour flow), device erosion, endocarditis, device thrombosis, thromboembolism, or stroke (Fig. 3). When subdividing the surgical group by defect complexity/additional procedures, no differences in any residual shunt were found (*p =* 0.45, Fig. 4).

**Table 4:** Predictors of residual shunt on multivariable analysis

Parameter	HR	95% CI	*p* value
Surgical approach	0.41	0.02–8.60	0.56
Age^a^	0.97	0.64–1.47	0.88
Male sex	0.39	0.04–4.04	0.43
Defect size	0.22	0.02–2.04	0.18
Complex defect	0.77	0.02–27.87	0.89

^a^Per 5 year age difference

*HR* Hazard ratio, *CI*, Confidence interval

Functional status was not significantly different between groups at follow-up, with a similar spread of follow-up NYHA status (*p* = 0.21). No patients suffered from headaches at follow-up. A single patient in the transcatheter group suffered a stroke following discharge from hospital secondary to device thrombosis. This patient has been followed by Haematology for a hypercoagulable state, which had previously resulted in multiple peripheral arterial interventions requiring femoral embolectomy, infra-inguinal bypass, and bypass graft thrombosis. Detailed results of patients at follow-up are presented in Table 5. A subgroup analysis of surgical patients was completed for those with simple defects (secundum defects and PFOs) compared with more complex, as well as those with isolated ASD repair compared with those receiving additional procedures. Predictably, cardiopulmonary bypass and cross clamp times were longer in repairs of complex defects and in multiple procedures. In the complex defect group, the median bypass time was significantly longer at 164 min (IQR 149–189 min) versus 112 min (IQR 99–127 min), *p* = 0.001. Cross-clamp time was also longer, with a median complex defect time of 103 min (IQR 81–123 min) versus 60 min (IQR 54–71 min), *p* < 0.001. This finding was consistent in the additional procedures group with median cardiopulmonary bypass times of 152 min (IQR 136–211 min) versus 112 min (99–135 min), *p* = 0.012. Cross-clamp times were similarly longer in those receiving additional procedures, with median times of 110 min (IQR 72–123 min) versus 61 min (54–72 min), *p* = 0.009. Although surgical times were consistently longer in both the complex defect and additional procedure surgical groups, significant complications were not detected.

**Table 5:** Follow-up outcomes^a^

		Transcatheter (*n* = 28)	Surgical (*n* = 33)	*p* value
Range (months)		0.5–56.5	0.25–89	
Follow-up (months), median		8.25	15	0.3
(IQR)		(2.5–23.25)	(2–52.5)	
Residual shunt		4 (14.3)	2 (6.1)	0.4
Residual Shunt >mild		0 (0)	0 (0)	
NYHA	1	23 (88.5)	31 (96.9)	0.21
	2	2 (7.7)	1 (3.1)	
	3	1 (3.9)	0 (0)	
	4	0 (0)	0 (0)	
Stroke		1 (3.6)	0 (0)	0.46
Headache		0 (0)	0 (0)	
Endocarditis		0 (0)	0 (0)	
Device erosion		0 (0)		
Device thrombosis		1 (3.6)		

^a^Reported as n(%) unless otherwise specified

*IQR* Interquartile range, *NYHA* New York Heart Association

## Discussion

Interest in minimally invasive options for ASD closure has increased with the advantage of better cosmesis in younger patients. Surgically, the right minithoracotomy and peri-areolar approaches in female patients allow avoidance of median sternotomy and reduced scar length. Our data shows that outcomes of transcatheter and minimally invasive surgical ASD repair are similar overall, with no significant difference seen in functional outcomes, headache, or amount of residual shunt. Although residual shunt in particular trended higher with transcatheter intervention, these tended to be mild, clinically insignificant shunts that did not result in a difference in functional status.

The number of peri-procedural complications was low in both groups and it is expected to be difficult to show a significant difference between the groups at small sample sizes. Similarly, showing a mortality difference between the two groups is also expected to require much larger sample sizes. As previously reported for other minimally invasive surgery, hospital and ICU length-of-stay was much shorter for transcatheter methods [6]. However, this immediate benefit was balanced by a trend toward higher residual shunt in the transcatheter group.

Late device migration or embolization is always a concern in transcatheter patients, although we did not see any such events in early follow-up. Delayed device erosion and embolization remains a possibility.

One patient in the transcatheter closure group experienced device thrombosis in the setting of multiple arterial thromboses and interventions. The importance of predisposition to arterial thrombosis is especially important in transcatheter closure and should be taken into account when selecting patients for device versus surgical closure.

Our centre uses an integrated, multidisciplinary ‘Heart Team’ approach in the evaluation of such patients, involving both cardiac surgery and interventional cardiology physician expertise. The collaborative nature of atrial septal defect closure at our institution allows for open discussion and facilitates optimal care. A ‘Heart Team’ approach is increasingly encouraged as the preferred method by which patient care decisions are made [15]. The closure approach selected is largely driven by patient-specific criteria outlined in the Methods section.

To the best of our knowledge, this is the first Canadian study directly comparing transcatheter and minimally invasive ASD closure and our data shows that both approaches can be used successfully. As with any new team-based approach, there is a learning curve that must be negotiated to develop a fruitful program however our data shows that overall complication rates are low with high procedural success rates.

## Limitations

Our observational study has a number of important limitations including limited sample size, single-centre data, and differences in follow-up duration. The small sample size increases the risk of Type 2 error. Also, it can lead to failure of detection of rare outcomes or complications. The possibility that a larger sample size could result in different comparative results between the two groups cannot be excluded.

With any observational study, the risk of systematic error is present. In our case, unavoidable selection bias exists, in that there are specific criteria for transcatheter ASD closure. Taking into account the limitations of a small sample size outlined above, this bias could lead to more favourable results in a larger population. Patients undergoing transcatheter closure were older and had a larger BMI but were otherwise comparable for baseline demographics. Notwithstanding the above, there remains the issue of unmeasured confounding inherent to the interpretation of non-randomized studies. Finally, referral bias may be a factor given that patients were selected from a single quaternary care centre.

## Conclusion

Overall, given the similar clinical outcomes of both groups, transcatheter and minimally invasive approaches to ASD closure are both safe and feasible in appropriately selected patients and represent attractive alternatives to traditional median sternotomy.

## Abbreviations

ASD: Atrial septal defect
AVSD: Atrioventricular septal defect
BMI: Body mass index
ICU: Intensive care unit
NYHA: New York Heart Association
PFO: Patent foramen ovale
TIA: Transient ischemic attack
TTE: Transthoracic echocardiography
